# Rapid molecular assays for detection of tuberculosis

**DOI:** 10.1186/s41479-018-0049-2

**Published:** 2018-05-25

**Authors:** Rkia Eddabra, Hassan Ait Benhassou

**Affiliations:** 1 Higher Institute of Nursing Professions and Health Techniques, Avenue Colonnel Major Habbouha Oueld Laâbid. Madinat Al Wahda I, Laayoune, Morocco; 2 Medical Biotechnology Center, Moroccan Foundation for Advanced Science, Innovation and Research (MAScIR), Rabat Design Center, Avenue Mohamed El Jazouli - Madinat Al Irfane, 10100 Rabat, Morocco

**Keywords:** Diagnosis, Drug resistance, *M. Tuberculosis*, Nucleic acid amplification tests, Sensitivity, Specificity, Whole-genome sequencing

## Abstract

Tuberculosis (TB) is an infectious disease that remains an important public health problem at the global level. It is one of the main causes of morbidity and mortality, due to the emergence of antibiotic resistant *Mycobacterium* strains and HIV co-infection. Over the past decade, important progress has been made for better control of the disease. While microscopy and culture continue to be indispensible for laboratory diagnosis of tuberculosis, the range of several molecular diagnostic tests, including the nucleic acid amplification test (NAAT) and whole-genome sequencing (WGS), have expanded tremendously. They are becoming more accessible not only for detection and identification of *Mycobacterium tuberculosis* complex in clinical specimens, but now extend to diagnosing multi-drug resistant strains. Molecular diagnostic tests provide timely results useful for high-quality patient care, low contamination risk, and ease of performance and speed. This review focuses on the current diagnostic tests in use, including emerging technologies used for detection of tuberculosis in clinical specimens. The sensitivity and specificity of these tests have also been taken into consideration.

## Background

Tuberculosis (TB), caused by *Mycobacterium tuberculosis*, is an infectious disease that poses a major global public health problem for both developing and developed countries. The World Health Organization (WHO) estimates that in 2015, 1.8 million people died from TB (including 0.4 million who were HIV-positive) [1]. In the same year, more than 95% of TB deaths occurred in low- and middle-income countries, and 170,000 children died of TB (excluding children with HIV) [1].

The usual site of TB infection is the lungs (pulmonary TB), but other organ systems can be involved (extrapulmonary TB) in spreading *M. tuberculosis*, including: pleural, lymphatic, urogenital, osteoarticular. The frequency of extrapulmonary disease increases with immune deficiency states, such as acquired immune deficiency syndrome patients (in whom extrapulmonary disease accounts for 50–60%) [2], or by the dissemination of *M. tuberculosis* throughout multiple organ systems (Miliary TB) [3]. Rapid and early diagnosis of TB and initiating optimal treatment would not only enable a cure of an individual patient but will reduce future numbers of TB cases [4].

The most widely used TB diagnostic test, microscopic examination of sputum for acid-fast bacilli (AFB), takes less than an hour; however, it is costly, lacks sensitivity and specificity, especially in HIV-infected individuals and children [5, 6]. Moreover, a positive result by this test does not discriminate between the *Mycobacterium* species [7]. Otherwise, Lowenstein-Jensen culture, generally used as the gold standard in suspected pulmonary cases, is more sensitive than smear microscopy, but it is time consuming (may take 4–8 weeks in solid media culture), and it requires adapted infrastructures and well-trained laboratory staff [8], which can delay effective medical interventions; therefore, the need for new rapid and accurate diagnostic methods has emerged. With the rapid evolution of molecular techniques, a wide variety of nucleic amplification tests (NAATs) such as polymerase chain reaction (PCR), real-time PCR, and loop-mediated isothermal amplification (LAMP), are available for the diagnosis of TB*.*

Currently, more than 50 new TB tests are in various stages of development [9]. Although the laboratory-developed and commercial NAATs assays have been primarily developed for the analysis of respiratory specimens, they are often used in non-respiratory specimens to the diagnosis of extra-pulmonary TB [10–12], because no commercial assay is approved for this purpose.

The present review summarizes the existing bibliography of molecular diagnostics tests for detection of TB. This is not an exhaustive review of all commercial NAATs; rather, the review presents the molecular techniques that have been used for detection of *M. tuberculosis* in clinical specimens. Some of them are already incorporated into the routine diagnostic, while other promising tests are still undergoing evaluation. .

### Rapid molecular tests incorporated into the routine diagnostic laboratory

#### COBAS TaqMan MTB

The qualitative COBAS TaqMan MTB (TaqMan MTB; Roche Diagnostics, Tokyo, Japan) test has also been introduced to replace the well-established COBAS Amplicor assay [13]. COBAS TaqMan MTB (CTM) test is a real-time PCR assay that amplifies part of the 16S rRNA gene with the use of a TaqMan probe for the detection of MTB complex DNA in clinical specimens. The turnaround time for analyzing 48 samples simultaneously using COBAS TaqMan is 2.5 h [14]. The COBAS TaqMan MTB assay is approved by the US Food and Drug Administration (FDA) for use in smear-positive and/or smear-negative pulmonary disease. The manufacturer’s instructions limit CTM application to respiratory specimens only [15]. However, many studies have evaluated the performance of the CTM assay for non-respiratory specimens [16–19].

The diagnostic accuracy of the CTM was poorer for the non-respiratory specimens than for the respiratory specimens. Bloemberg et al. [17] examined 838 respiratory specimens and found Cobas TaqMan MTB assay to have 88.4% sensitivity and 98.8% specificity, compared to a sensitivity of 63.6% and a specificity of 94.6% for the 305 non-respiratory specimens. While using culture as the golden standard for all specimens, the sensitivity and specificity was 82.4% and 97.7% respectively.

Studies have found that the Cobas assay had higher sensitivity in smear-positive specimens than in smear-negative specimens [20], which might be attributable to the decontamination and concentration steps [21]. The sensitivity of the assay ranges from 96.9% to 98% in smear-positive samples and from 34.9% to 79.5% in smear-negative samples, while the specificity ranges from 78.1% to 100% in smear-positive samples and from 98.7% to 99% in smear-negative samples [17–19, 22–24]. However, these results vary from study to study. Some studies have suggested that this variance is due to the acid-fast bacilli (AFB) smear status, variable specimen types and incidence of TB [19, 20]. The results of the Cobas TaqMan MTB assay should be carefully interpreted alongside the clinical data.

#### Loop-mediated isothermal amplification

Loop-mediated isothermal amplification (LAMP) (Eiken Chemical Co. Ltd., Tokyo, Japan) assay is an isothermal molecular method developed by Notomi et al. [25]. LAMP has been successfully implemented in nucleic acid research, and in clinical application as a screening tool [26]. Several LAMP-based assays have been developed to detect *M. tuberculosis* infection, targeting *gyrB* [27], *rrs* [28], *rimM* [29], *IS6110* [30], *hspX* [31], *mpb64* [32] and *sdaA* gene [33].

LAMP is an isothermal nucleic acid amplification technique, in which amplification is carried out at a constant temperature without the need for a thermal cycler. This method amplifies very few copies of target DNA with high specificity, efficiency, and rapidity under isothermal conditions using a set of 4 specially designed primers and a DNA polymerase with strand displacement activity [25, 34, 35]. LAMP was recommended by WHO in August 2016 for diagnosing pulmonary TB in adults as a potential replacement for smear microscopy.

Many studies show that LAMP offers potential advantages over PCR for its simplicity, speed, specificity and cost-effectiveness. These studies favor its use in simplified testing systems, which could be appropriate in settings with limited resources [2, 35].

TB-LAMP has higher sensitivity for smear-positive samples (92.1%–100%) than for smear-negative samples (52.1%–90.3%) [36–39]. For extrapulmonary samples, a recent study found that LAMP had a good sensitivity (95.6%) compared to 3 conventional methods: liquid culture, solid culture, and smear microscopy (69.6%, 65.2% and 17.4%, respectively) [40]. It has been observed that the exposure of reaction tubes to aerosol contamination was identified as one of the possible causes of false-positives results [29].

In the policy guide, WHO excluded all data obtained from extra pulmonary samples, and the validation of TB-LAMP testing with extra pulmonary samples is still under investigation [41]. To date, LAMP has not been fully evaluated in HIV patients and children (no data have been published for children samples).

#### Gene Xpert TB assays

Xpert MTB/RIF (Xpert; Cepheid Inc., Sunnyvale, California, United States of America [USA]) is an automated polymerase chain reaction (PCR) test utilizing the GeneXpert platform [42, 43]. The Xpert MTB/RIF assay detects MTB and rifampicin resistance within two hours of starting the test, with minimal hands-on technical time [44]. It has been approved by the WHO and the US (FDA) (Table 1) [45–47]. The test procedure may be used directly on clinical specimens, either raw sputum samples or sputum pellets, and samples created after decontaminating and concentrating the sputum [42]. Several studies reported that Xpert MTB/RIF is a sensitive method for rapid diagnosis of TB, compared to conventional techniques [48, 49].

**Table 1:** Characteristics of approved (WHO and/or FDA) molecular assays for rapid detection of MTB and drug-resistance

Test specification	COBAS TaqMan MTB	TB-LAMP	Xpert MTB/RIF	XpertMTB/RIF Ultra	GenoType MTBDR*plus*	GenoType MTBDR*sl*
Manufacturer	Roche Diagnostics	Eiken Chemical Co	Cepheid	Cepheid	Hain Lifescience	Hain Lifescience
Technology	Real-time PCR	Loop-mediated Isothermal amplification (LAMP)	Real-time PCR (molecular beacons)	Real-time PCR (molecular beacons)	Multiplex PCR + reverse hybridization	Multiplex PCR + reverse hybridization
Detects	MTB	MTB	MTB + RIF resistance	MTB + RIF resistance	MTB + resistance to RIF and INH	MTB diagnosis + resistance to FLQ and SLID
Target	16S rRNA	*IS6110*	*rpoB* gene	*rpoB* gene	*rpoB, katG, inh* genes	*gyrA, rpo, rrs, eis* genes
Time to results	2.5 h	< 1 h [41]	2 h [43]	< 90 min [58]	5 h	5 h
Approval status	FDA	WHO	WHO	WHO	WHO	WHO
Recommandations	As a confirmatory test, on smear-positive specimens	As a replacement for smear microscopy in adults or a follow-on test to smear microscopy in adults (when further testing of sputum smear-negative specimens is necessary) [41]	As the initial diagnostic test for tuberculosis in patients (adults and children) suspected of having active TB disease with either multidrug-resistant TB or HIV-associated TB [47]	As a replacement of Xpert MTB/RIF due to its greater sensitivity in detecting MTB [66]	As the initial test instead of phenotypic culture-based DST to detect resistance to RIF and INH, in persons with a sputum smear-positive specimen or a cultured isolate of MTBC, from both pulmonary and extrapulmonary sites [73].	For patients with confirmed RIF-R TB and/or MDR TB as the initial test, instead of phenotypic culture-based DST, to detect resistance to FLQ and SLID [83]
Benefits	− Does not require expensive instruments and laboratory environments	- Requires minimal expertise - Speed and cost effective	- Requires minimal expertise - Excellent sensitivity in tests of smear-positive sputum samples	− Improve detection of mutants at codon 533 [58] − Differentiate silent mutations at codons 513 and 514 [58]. - Detect a hetero-resistant sample [58]	- High sensitivity for detection of RIF resistance.	- High sensitivity and specificity for detection of FLQ and SLID resistance [84].
Limitations	- Exhibits heterogeneous performance from study to study [24] - Does not screen for any markers of drug resistance	− Requires several manual steps. − Inferior performance in smear negatives sputum samples [102] − False positives results due to aerosol contamination [29] − Does not screen for any markers of drug resistance	- High costs and sophisticated hardware. - Low sensitivity in smear-negative pulmonary samples and special populations (HIV-positives, children, extrapulmonary TB). - Do not accommodate all mutations conferring resistance to anti-TB agents [58]	− Low specificity in patients with a recent history of TB treatment or from high-incidence countries [61]. − Should not be used as a replacement test for culture in children [65] − Do not accommodate all mutations conferring resistance to anti-TB agents [61]	− Over diagnose the presence of MTB complex DNA in culture-negative samples [81]. − Low sensitivity to detect INH resistance. − Complexity, the number of steps. − Requires training for interpretation of results − Do not perform well when applied to paucibacillary clinical specimens − Do not accommodate all mutations conferring resistance to anti-TB agents	− Not optimal to detect resistance to KAN [84] and ethambutol [86]. − Low sensitivity in smear-negative samples [76]. − Do not accommodate all mutations conferring resistance to anti-TB agents [84].
Price per test	Not available	US$ 6	US$ 9.98	US$ 9.98	US$10	US$10

US$: United States dollars

hrs: hours

min: minutes

Studies evaluating Xpert performance in pulmonary and extrapulmonary samples in low and intermediate prevalence settings [49, 50], showed a sensitivity ranging from 47.8% to 73% and from 28.2% to 73.2% for smear-negative pulmonary specimens and smear-negative extrapulmonary specimens, respectively. The sensitivity of Xpert MTB/RIF in smear-positive samples was 100% [49, 50]. The Xpert MTB/RIF assay is less sensitive than liquid cultures for the detection of MTB in both children and adults [51, 52]. Xpert has generally performed very well as a rapid test for rifampicin resistance (RIF-R), with a pooled sensitivity and specificity of 94% and 98%, respectively [44] (Table 2). However, the ability of the assay to detect the RIF-R in a sample with mixtures of RIF-susceptible and RIF-sensitive *M. tuberculosis* populations is dependent on the type of mutation present [42].

**Table 2:** Sensitivity and specificity of endorsed molecular assays for rapid detection of drug-resistant TB

Assay	Detection of drug resistance	Sensitivity % (95% confidence interval)	Specificity % (95% confidence interval)	References
Xpert MTB/RIF	RIF	95 (90–97)	98 (97–99)	44*
Xpert Ultra	RIF	92.7 (80.1–98.5)	98 (92.8–99.9)	58*
GenoType MTBDR*plus*	RIF	Smear positive sample: 88.2 (72.6–96.7)	Smear positive sample: 89.5 (75.2–97.1)	77*
		Smear negative and culture positive direct sample: 100 (29.2–100)	Smear negative and culture positive direct sample: 63.6 (30.8–89.1)	
	INH	Smear positive sample: 91.7 (77.5–98.3)	Smear positive sample: 97.2 (85.5–99.9)	
		Smear negative and culture positive direct sample: 60 (14.7–94.7)	Smear negative and culture positive direct sample: 100 (66.4–100)	
	MDR-TB (RMP&INH)	Smear positive sample: 96.4 (81.7–99.9)	Smear positive sample: 100 (88.8–100)	
		Smear negative and culture positive direct sample: 100 (15.8–100)	Smear negative and culture positive direct sample: 100 (47.8–100)	
GenoType MTBDR*sl*	FLQ	100 (95.8–100)	98.9 (96.1–99.9)	84*
	AMK	93.8 (79.2–99.2)	98.5 (95.5–99.7)	
	KAN	89.2 (79.1–95.6)	98.5 (95.5–99.7)	
	CPM	86.2 (68.3–96.1)	95.9 (92.2–98.2)	

44*: reference standard was phenotypic culture-based DST

58*: results were compared with phenotypic susceptibility testing and Xpert MTB/RIF

77*: results were compared with the conventional liquid culture based reference standard method, BACTEC MGIT 960 culture and DST

84*: results were compared with phenotypic DST

Several studies have found that Xpert MTB/RIF was not capable of detecting resistance-conferring mutations located outside the 81 bp rifampicin resistance determining region (RRDR) of the *rpoB* gene [42, 53]. Results obtained in Swaziland show that the Xpert MTB/RIF assay did not detect the *rpoB* I491F mutation in 38/125 (30%) of multidrug-resistant strains, as compared to DNA sequencing [53]. The high frequency of the I491F mutation highlights the limits of the assay. Thus, it is important to detect this mutation and complement commercial assays for the diagnosis of RIF-R *M. tuberculosis* in routine conditions, particularly in countries where this specific mutation is frequent [54].

The second limitation of Xpert MTB/RIF compared to sequencing methods is that Xpert can not differentiate silent mutations emerging at various positions in the RRDR of the *rpoB* gene [55]. These missed mutations within the RRDR, together with those outside the RRDR, may cause misinterpretation of RIF susceptibility, rendering treatment ineffective and may be untraceably circulated through chains of transmission.

Luetkemyer et al. and Parcell et al. [56, 57] showed in their studies that the performance of Xpert MTB/RIF did not differ between higher- and low-prevalence areas. For HIV-associated TB, Xpert MTB/RIF has lower sensitivity [44].

To improve the sensitivity and specificity of the current assay in detection of TB and RIF-R, respectively, a new version of the Xpert MTB/RIF assay, called Xpert Ultra, has been developed. The Xpert MTB/RIF Ultra was designed by adding two amplification targets (IS*6110* and IS*1081*), 25 different RRDR mutations covering almost the entire *rpoB* RRDR from codons 510 to 533, doubling the size of the DNA delivered to PCR reaction, and other technical enhancements to reduce the limits of detection from 112.6 CFU/mL of sputum for Xpert to 15.6 CFU/mL of sputum for Ultra [58].

In 2015, Alland et al. [59] found that Xpert MTB/RIF Ultra is much more sensitive than Xpert, and is likely to be as sensitive as liquid TB culture. The multi-center study (1520 person with signs or symptoms of pulmonary TB) carried out by the Foundation for Innovative New Diagnostics (FIND) [60] revealed that compared to culture the sensitivity of Ultra was 5% higher than that of Xpert MTB/RIF (87.8% vs 82.9%), but the specificity was 3.2% lower (94.8% vs 98%). In the same study, the sensitivity of Ultra was 17% higher than Xpert MTB/RIF in people with smear-negative, culture positive TB (61.3% vs 44.5%) and 12% higher in HIV-infected patients (87.8% vs 75.5%).

The higher sensitivity of Ultra is accompanied by a loss of specificity, particularly among individuals with a history of previous TB treatment [61]. Arend and van Soolingen [62] reported that the excess of false positive Xpert Ultra results found by Dorman et al. [61] can be explained by detection of DNA from non-viable *M tuberculosis*, a phenomenon previously shown for Xpert MTB/RIF [63].

In a study of 378 children, Ultra’s sensitivity was 24% higher than that of MTB/RIF [64]. A recent study performed in South African children (367 children) hospitalized with suspected pulmonary TB, has shown that Ultra detected 75.3% of culture-confirmed cases. The authors concluded that Ultra should not be used as a replacement test for culture in children [65].

The rates of detection of RIF susceptibility were comparable between Xpert and Ultra [58]. Ultra improved detection of mutants at codon 533, differentiated silent mutations at codons 513 and 514, and detected a hetero-resistant sample that was missed by both phenotypic susceptibility testing and Xpert [58]. However, mutations such as IIe491Phe are not detected by Xpert Ultra [61].

At the end of March 2017, the WHO recommended the replacement of Xpert by Xpert MTB/RIF Ultra, based on its increased sensitivity compared to Xpert, which could improve the diagnosis of paucibacillary forms of TB disease such as childhood TB, HIV-associated TB, or extrapulmonary TB [66].

Cepheid is also slated to release another major technology improvement called the GeneXpert Omni for point-of-care testing for TB and rifampicin resistance, using the same cartridges as those used in the current GeneXpert machine. GeneXpert Omni is a portable single-cartridge testing unit, less expensive than the current Genexpert, and has four hours of battery life. Because of the above-mentioned characteristics, it is very useful, particularly in remote settings where very limited infrastructure is available for rapid diagnosis of TB. The projected release of the Omni in emerging markets is at the end of 2018, and it has yet to be launched or evaluated by the WHO [67].

Cepheid Inc. is also currently developing another cartridge, the Xpert XDR, which will provide resistance to isoniazid, fluoroquinolones and aminoglycosides [68]. Xpert XDR may be highly useful for extensively drug-resistant tuberculosis (XDR-TB) triaging in high DR-TB settings, considering the new fluoroquinolone-based short regimens [69, 70].

#### GenoType Line-Probe Assays

Molecular detection of *M. tuberculosis* by line probe assays (LPA) was introduced in 1995. The assay also allows for rapid detection of drug resistance [71]. LPA, known as solid-phase hybridization assays, involves a series of steps including: extraction of DNA from cultures or directly from clinical samples, PCR amplification of nucleic acid sequences, denaturation, hybridization of the biotinylated PCR amplicons with oligonucleotide probes immobilized on a strip and colorimetric development that allows for lines to be seen where the probes are located [72]. Some of these LPA tests are INNO-LiPA Mycobacteria (Innogenetics, Belgium) for the distinction of the *M. tuberculosis* (sub) species and the most frequently encountered nontuberculous mycobacteria, and Genotype MTBDR*plus* and GenoType MTBDR*sl* (Hain LifeScience GmbH, Nehren, Germany) for rapid detection of MTB and its associated drug resistance, as discussed above.

The Genotype *M. tuberculosis* drug resistant (MTBDR) *plus* (Version 2.0) is a qualitative in vitro test for detection of the *M. tuberculosis* complex and simultaneous detection of mutation in the *rpoB* and *katG* genes for rifampicin (RIF) and isoniazid (INH) resistance, respectively, and its use is approved by WHO [73]. This test can be used on bacterial cultures or smear-positive clinical specimens and takes approximately 5.5 h to perform [74]. Many studies confirm that the diagnostic performance of Genotype MTBDR*plus* (Version 2.0) LPA for detection of multidrug-resistant tuberculosis (MDR-TB) in direct smear-positive sputum sample was highly sensitive and specific [75–77]. However, the sensitivity of the assay should be improved for detection of MDR-TB in direct smear-negative sputum specimens [77].

In a study conducted on 242 multidrug-resistant and 30 pansusceptible *M. tuberculosis* isolates, the performances of the LPA and DNA sequencing in detecting RIF and INH resistance-associated mutations were compared to that of a conventional agar proportion DST. The results show that the sensitivity for detection of MDR-TB was 78.5% with the GenoType MTBDR*plus* test and 91.3% by resistance gene sequencing [78]. The specificity for RIF resistance, INH resistance, and MDR-TB detection was 100% by both methods. However, DNA sequencing demonstrated superior performance in detecting INH resistance. The study suggested that additional alleles associated with INH resistance should be evaluated to improve the sensitivity of the GenoType MTBDR*plus* test.

In terms of diagnosis, a number of studies have demonstrated that GenoType MTBDR*plus* (Version 2.0) present greater sensitivity for detection of MTB complex DNA in smear-positive samples [79–81]. Barnard et al. [81] demonstrated that GenoType MTBDR*plus* (Version 2.0) overdiagnoses the presence of *M. tuberculosis* complex DNA in culture-negative samples, which may be explained by the amplification of DNA released from nonviable bacilli, by laboratory cross-contamination, or by a transcription error. Further research evaluating the effect of smear status, smear grade and other covariates such as HIV on the diagnostic accuracy of GenoType MTBDR*plus* (Version 2.0), for detection of *M. tuberculosis* complex DNA is needed.

Another LPA, the GenoType MTBDR*sl* 2.0 (Hain LifeScience GmbH, Nehren, Germany) line probe assay was developed for the detection of *M. tuberculosis* and simultaneous detection of resistance-conferring mutations of fluoroquinolones (FLQ) (*gyrA* and *gyrB* genes) and second-line injectable drugs (SLID) (*rrs* and *eis* genes) [82]. The target region for detection of ethambutol (EMB) (a first-line anti-tuberculosis drug) resistance (*embB* codon 306), present in MTBDR*sl* v1, has been removed from v2.0. WHO recommended the use of the GenoType MTBDR*sl* 2.0 assay as an initial test, instead of phenotypic culture-based drug susceptibility testing (DST), to detect FLQ and SLID resistance in confirmed RIF-R and MDR patients [83].

Gardee and colleagues [84] reported that GenoType MTBDR*sl* 2.0 has shown an improvement in sensitivity and specificity for the determination of molecular resistance to both FLQ (100% and 98.9%) and SLID (89.2% and 98.5%) (Table 2). The same study confirmed the presence of *gyrA* mutations missed by the assay, which were detected by whole-genome sequencing (WGS).

It has been reported by several authors [85, 86] that MTBDR*sl* v1 showed poor accuracy for detecting resistance to EMB (55% and 71%) compared to FLQ and SLID. Only mutations covered by wild-type or mutant probes can be detected. Other mutations are required to be targeted by the assay to increase sensitivity and specificity.

### Later-stage or marketed tuberculosis diagnostic test candidates

Several new diagnostics are emerging from the development pipelines, and currently more than 50 new TB tests are in various stages of development [9]. The majority of the tools in the pipeline are still in early stages of development and/or evaluation. A few new technologies are available on the market where the data are unavailable or limited [87]. Among the assays marketed without any/or a few data published are EasyNAT TB (Ustar Biotechnologies, Hangzhou, China), FluoroType MTB (Hain Lifesciences GmbH, Nehren, Germany) and PURE-LAMP (Eiken Chemical Company, Japan) (Table 3).

**Table 3:** characteristics of molecular assays for rapid detection of MTB and drug-resistance, not yet approved by WHO and/or FDA

Test specification	PURE TB-LAMP	FluoroType MTB	EasyNAT TB	Xpert Omni	Xpert XDR
Manufacturer	Eiken Chemical Co	Hain Lifescience	Ustar	Cepheid	Cepheid
Technology	Procedure for Ultra Rapid Extraction Loop-mediated isothermal amplification	Real-time PCR (HyBeacon fluorescence)	Isothermal DNA amplification Lateral flow	Real-time PCR (molecular beacons)	Real-time PCR (molecular beacons)
Detects	MTB	MTB	MTB	MTB + RIF resistance	MTB + resistance to INH, FLQ and SLID
Time to results	90 min [90]	3 h	90 min	∼ 110 min [67]	90 min
Current status	The assay is now on path for WHO review	CE-IVD marked	CE-IVD certified Approved by China FDA	Platform under development. Launch expected at the end of 2018.	Assay under development.
Benefits	− Performed quickly [36]. − No sophisticated laboratory equipment is needed [36]. − Requires fewer procedures and consumables. − Sensitivity higher than smear microscopy [36]	− Fluorocycler system is suitable for low number of samples or for large series [92]. − Sensitivity equivalent to other molecular tests.	− Requires basic laboratory equipment	− Point of care − Performed quickly [95].	Assay under development.
Limitations	− Yield false-negative results [90] − Possible risks for cross contamination. − Does not screen for any markers of drug resistance. − Further studies are required [98].	− Low specificity due to DNA contamination [94] − Low sensitivity in smear-negative [92]. − Does not screen for any markers of drug resistance. - Further studies are required [92].	− Not suitable for diagnosis of extrapulmonary TB [100]. − Does not screen for any markers of drug resistance.	− Processes one sample at a time [95] − Cost and accessibility will limit adoption in high-endemic areas − Do not accommodate all mutations conferring resistance to anti-TB agents	Assay under development.
Price per test	Not available	Not available	US$ 6–8	Not available	Not available

#### Pure-LAMP

The PURE (Procedure for Ultra Rapid Extraction)-LAMP (Eiken Chemical, Tokyo, Japan) is a manual TB detection tool based on loop-mediated isothermal amplification (LAMP) using a nucleic acid amplification method, developed from 2007 to 2010, by Chemical Company and FIND. LAMP with the PURE test can be performed quickly (within 90 min) and includes three steps, sample preparation, amplification with LAMP and visual detection of fluorescence light from the reaction tube using UV light [36, 88].

Several studies showed that PURE-LAMP has very high sensitivity and specificity, which makes it economic, cost effective and rapid method for the diagnosis of tuberculosis [8, 89]. Mitarai et al. [88] reported the sensitivity of PURE-LAMP in smear-negative TB patients to be 55.6%, and 98.2% among smear positive TB patients. Ou et al. [36] reported that the sensitivity of the PURE-LAMP in smear-negative TB patients and culture-positive TB patients was 53.81%, the overall sensitivity was 70.67% and the specificity of PURE-LAMP was 98.32%. Kouzaki et al. [90] showed that PURE-LAMP may potentially be a valuable tool for the diagnosis of extrapulmonary TB. N’guessan et al. [8] compared the performances of sputum smear microscopy (SSM) after Ziehl-Neelsen staining and PURE TB-LAMP assay. The results of this study show that the sensitivity of TB-LAMP assay is higher than SSM. However, SSM specificity was higher than molecular method. Thus, PURE-LAMP is recommended along with other diagnostic methods to verify the diagnosis of TB, particularly in false-negative samples [90]. The assay is now on path for WHO review.

#### FluoroType MTB

FluoroType MTB (Hain Lifescience, Nehren, Germany) assay is a rapid molecular diagnostic test using real-time PCR to detect *M. tuberculosis* complex from respiratory and non-respiratory clinical specimens. The FluoroType MTB test is based on the HyBeacon fluorescence technology [91]. The amplification is performed on the FluoroCycler instrument (Hain Lifescience) while the detection is performed by melt curve analysis (MCA) with single stranded oligonucleotides labeled with fluorescent dyes that are complementary to the amplified DNA. The method gives results for multiple specimens within 3–4 h [92].

The first evaluation study of the new FluoroType MTB assay performed for the direct detection of *M. tuberculosis* in clinical respiratory tract specimens demonstrated that sensitivity and specificity were 95.1% and 96.4%, respectively, in 661 specimens tested, and that sensitivity was 100% for smear-positive and 84.6% for smear negative specimens, respectively [93]. Hofmann-Thiel and Hoffmann [92], found that FluoroType MTB assay had a sensitivity of 88.1% (smear-positive 100%; smear-negative 56.3%) and a specificity of 98.9%, in comparison with culture. The authors concluded that the test results were similar to non-nucleic acid amplification tests on the market, and that the Fluorocycler system is suitable for low numbers of samples.

Recently, the system has been evaluated in resource-poor settings [94]. The diagnostic accuracy of the FluoroType MTB assay was calculated using solid culture as the reference standard and described by light-emitting diode fluorescence smear positivity, HIV status and Xpert MTB/RIF. The authors found that FluoroType MTB has a sensitivity equivalent to other molecular tests and identified more culture-positive samples than Xpert MTB/RIF, but its specificity was lower than expected, due to DNA contamination during the sample preparation steps.

The assay is marketed in Europe and launched for marketing in April 2017, however it is not yet evaluated by WHO [95].

#### EasyNAT TB

The EasyNAT TB isothermal nucleic acid amplification diagnostic kit (Ustar Biotechnologies Co. Ltd., Hangzhou, China) uses isothermal cross-priming amplification technology for the qualitative detection of *M. tuberculosis* [96]. The assay was approved in 2014 for detection of pulmonary TB by the China FDA [97]. To date, EasyNAT TB has been evaluated only for the detection of pulmonary TB in adults [98, 99].

Ou et al. and Mhimbira et al. [98, 99] reported sensitivity for MTB detection against culture of 84.1% and 66.7%, and specificity of 97.8% and 100%. Sensitivity in these studies was further reduced when only smear-negative but culture-positive pulmonary TB cases were tested (59.8% and 10%), suggesting further evaluation in larger study populations from different regions that are endemic for TB [99].

Bholla et al. [100] evaluated the performance of EasyNAT for diagnosis of extrapulmonary TB (tuberculous lymphadenitis of children), and found that the sensitivity and specificity was 19% and 100%, respectively. The authors concluded that EasyNAT is not suitable for diagnosis of extrapulmonary TB.

#### Rapid whole-genome sequencing

Early detection of drug resistance is crucial in choosing the most effective treatment to avert mortality of infected individuals and to prevent the risk of transmission of drug-resistant TB [101]. Molecular tests such as quantitative real time amplification (e.g. Xpert MTB/RIF) and line probe assays (e.g. GenoType MTBDRplus*/sl*), although more rapid (less than a day), are able to identify only limited numbers of specific resistance mutations in drug target genes [102–104]. Both technologies lack capacity to detect mutations outside of the rifampicin resistance-determining region (RRDR) of the *rpoB* gene (e.g. I491F mutation) [53, 105] or to differentiate silent mutations from those that effect drug efficacy (e.g. the substitution of CGT for TTG in codon 533 of the *rpoB* gene), leading to false positive results [106, 107]. The ability to detect and identify such mutations among patients with TB has become necessary, and would be of tremendous value in quickly guiding the initiation of appropriate therapy. Genome sequencing has the potential to overcome these problems and can provide clinically relevant data within a time frame that can influence patient care.

The past decade has seen a considerable expansion of sequencing capacity improving its availability for routine laboratories. Whole-genome sequencing (WGS) has been shown to provide a rapid and comprehensive view of the genotype of *M. tuberculosis*, and allows simultaneous identification of all known resistance-associated loci with high concordance to culture-based drug susceptibility testing (DST), while also providing opportunities to characterize other loci as predictive of resistance or not [108]. Results reported by Shea et al. [109] for 462 prospectively collected *M. tuberculosis* complex strains, show that strain identification by WGS was determined to be 99% accurate, and concordance between drug resistance profiles generated by WGS and culture-based DST methods was 96% for 8 drugs (RIF, INH, FLQ, PZA, KAN, EMB, STR, ETH), with an average resistance-predictive value of 93% and susceptible-predictive value of 96%. Furthermore, WSG can be used in outbreak surveillance to understand transmission in a population [110].

Despite the perceived benefits of WSG for routine diagnosis and management of drug-resistant TB, it has only been implemented in a few high-income countries, low-TB burden settings such as England [111]. The implementation of WGS in the clinical setting is hindered by some limitations, including the requirement of bacterial enrichment by culturing, prior to DNA isolation and sequencing, and this generally takes at least a couple of weeks. Limited studies demonstrated the use of WSG to generate results within a shorter turnaround time. Findings from studies performed by Brown et al. [112] and Nimmo et al. [108] showed that WGS can be successfully performed directly from uncultured sputa.

The complexity of WGS data and its analysis also represents a significant challenge, pointing to the need of bioinformatics expertise among clinical microbiologists [111]. A number of groups are now developing software to help people without bioinformatic skills to process and analyse large sets of raw data. In this context, Coll et al. [113] published an exhaustive library with 1325 mutations predictive of DR for 11 anti-tuberculosis drugs (AMK, CAP, EMB, ETH, INH, KAN, MOX, OFX, PZA, RMP and STR) and developed an online tool that rapidly analyses raw sequence data and predicts resistance. However, further work will be required to clarify the current discrepancies between genotype and phenotype [114], as well as the understanding of the genetic basis of antibiotic resistance, which complicates the interpretation of WGS data [111].

## Conclusions

A number of studies have highlighted the role of rapid molecular diagnostic in diagnosis, management and monitoring of TB. Compared with traditional testing methods, molecular TB diagnostics have been shown to reduce the turnaround time (the results can be obtained within hours from receipt of the specimen) with reliable results on smear-positive specimens, but have lower sensitivities especially in specimens that are negative by microscopy (and are generally less effective in children compared with adults). Furthermore, the additional cost, the need for a laboratory infrastructure (i.e. Xpert MTB/RIF), as well as the need for technicians trained in molecular techniques are limitations that pose considerable challenges, especially in low-resource settings. Despite significant advances in the development of novel tests, molecular tests cannot replace culture, but should be used in addition to conventional tests (smear microscopy, culture, and phenotypic drug susceptibility testing) and clinical data for TB diagnosis, as highlighted by other studies [115, 116].

Many questions remain concerning the effectiveness of NAATs for smear-negative pulmonary and extrapulmonary TB in adults, detection of paucibacillary forms of TB (e.g. pediatric disease) and previously treated individuals. Furthermore, no tests are available which are universally applicable to all patients.

Whole genome sequencing (WGS) has the potential to revolutionize the diagnosis of *M. tuberculosis* infection. However, the utility of WSG is currently limited due to the major drawbacks of sequencing, such as the costs associated with the test, the technical skill required, complex bioinformatic procedures and the unavailability of sequencing facilities. There are currently no plans for routine implementation of WSG in resource-limited, high-TB burden countries.

Research work must continue towards developing new molecular and advanced techniques for rapid and accurate diagnosis of TB, with better performance characteristics, that can be easily implemented for routine TB diagnosis in low-resource countries.

## Abbreviations

AFB: Acid-fast bacilli
AMK: Amikacin
CAP: Capreomycin
CE-IVD: European Conformity-in vitro diagnostic
CTM: COBAS TaqMan
DNA: Deoxyribonucleic acid
DST: Drug susceptibility testing
EMB: Ethambutol
ETH: Ethionamide
FDA: Food and Drug Administration
FIND: Foundation for Innovative New Diagnostics
FLQ: Fluoroquinolones
HIV: Human immunodeficiency virus
INH: Isoniazid
KAN: Kanamycin
LAMP: Loop-mediated isothermal amplification
LPA: Line probe assay
MCA: Melt curve analysis
MDR: Multidrug-resistant
MOX: Moxifloxacin
MTB: *Mycobacterium tuberculosis*
NAATs: Nucleic acid amplification tests
OFX: Ofloxacin
PCR: Polymerase chain reaction
PZA: Pyrazinamide
QRDR: Quinolone resistance-determining regions
RIF: Rifampicin
RIF-R: Rifampicin resistance
RRDR: Rifampicin Resistance Determining Region
RT-PCR: Reverse transcriptase-polymerase chain reaction
SLID: Second-line injectable drug
SSM: Sputum smear microscopy
STR: Streptomycin
TB: Tuberculosis
WGS: Whole-genome sequencing
WHO: World Health Organization
XDR: Extensively drug-resistant

## References

[1] World Health Organization (WHO). In: WHO, editor. Global Tuberculosis Report 2016. Geneva; 2016.

[2] García-Elorriaga G, del Rey-Pineda G. Practical and laboratory diagnosis of tuberculosis from sputum smear to molecular biology. SpringerBriefs in Microbiology. 2015; 10.1007/978-3-319-20478-9.

[3] Tortoli E (2014). Microbiological features and clinical relevance of new species of the genus *Mycobacterium*. Clin Microbiol Rev.

[4] Dye C (2012). The potential impact of new diagnostic tests on tuberculosis epidemics. Indian J Med Res.

[5] Mugusi F, Villamor E, Urassa W, Saathoff E, Bosch RJ, Fawzi WWHIV (2006). Co-infection, CD4 cell counts and clinical correlates of bacillary density in pulmonary tuberculosis. Int J Tuberc Lung Dis..

[6] Dunn JJ, Starke JR, Revell PA. Laboratory diagnosis of *Mycobacterium tuberculosis* infection and disease in children. J Clin Microbiol. 2016; 10.1128/JCM.03043-15. 10.1128/JCM.03043-15 PMC4879301 26984977

[7] Dezemon Z, Muvunyi CM, Jacob O. Staining techniques for detection of acid fast bacilli: what hope does fluorescein-diacetate (FDA) vitality staining technique represent for the monitoring of tuberculosis treatment in resource limited settings. Int Res J Bacteriol. 2014;1(1)

[8] N’guessan K, Adegbele J, Coulibaly I, Kouame-N’takpé N (2017). Clinical performances of Pure TB-Lamp kit for *M. tuberculosis* complex detection in sputum samples. J Tuberc Res.

[9] World Health Organization (WHO) (2015). Global tuberculosis report 2015.

[10] Caws M, Wilson SM, Clough C (2000). Role of IS6110-targeted PCR, culture, biochemical, clinical, and immunological criteria for diagnosis of tuberculous meningitis. J Clin Microbiol.

[11] O'Sullivan CE, Miller DR, Schneider PS, Roberts GD (2002). Evaluation of gen-probe amplified *Mycobacterium tuberculosis* direct test by using respiratory and non respiratory specimens in a tertiary care centerlLaboratory. J Clin Microbiol.

[12] Pai M, Flores LL, Pai N, et al. Diagnostic accuracy of nucleic acid amplification tests for tuberculous meningitis: a systematic review and meta-analysis*.* Lancet Infect Dis 2003;3:633–3. 10.1016/s1473-3099(03)00772-2 14522262

[13] Leylabadlo HE, Kafil HS, Yousefi M, Aghazadeh M, Asgharzadeh M (2016). Pulmonary tuberculosis diagnosis: where we are?. Tuberc Respir Dis.

[14] Palomino JC (2009). Molecular detection, identification and drug resistance detection in *Mycobacterium tuberculosis*. FEMS Immunol Med Microbiol.

[15] Roche Diagnostics GmbH. COBAS TaqMan MTB Test. Available from: http://tbevidence.org/documents/rescentre/sop/MTB%20TaqMan%20PI.pdf#search=‘cobas+taqman+mtb’. Accessed on 3rd July 2015.

[16] Kim JH, Kim YJ, Ki CS, Kim JY, Lee NY (2011). Evaluation of Cobas TaqMan MTB PCR for detection of *Mycobacterium tuberculosis*. J Clin Microbiol.

[17] Bloemberg GV, Voit A, Rittera C, Deggim V, Böttgera EC (2013). Evaluation of Cobas TaqMan MTB for direct detection of the *Mycobacterium tuberculosis* complex in comparison with Cobas Amplicor MTB. J Clin Microbiol.

[18] Lee MR, Chung KP, Wang HC, Lin CB, Yu CJ, Lee JJ, Huseh PR (2013). Evaluation of the Cobas TaqMan MTB real-time PCR assay for direct detection of *Mycobacterium tuberculosis* in respiratory specimens. J Med Microbiol.

[19] Jönssona B, Lönnemark E, Ridell M (2015). Evaluation of the Cobas TaqMan MTB test for detection of *Mycobacterium tuberculosis* complex. Infect Dis (Lond).

[20] Huh HJ, Koh WJ, Song DJ, Ki CS, Lee NY (2015). Evaluation of the Cobas TaqMan MTB test for the detection of *Mycobacterium tuberculosis* complex according to acid-fast-bacillus smear grades in respiratory specimens. J Clin Microbiol.

[21] Park KS, Kim JY, Lee JW, Hwang YY, Jeon K, Koh WJ, Ki CS, Lee NY (2013). Comparison of the Xpert MTB/RIF and COBAS TaqMan MTB assays for detection of *Mycobacterium tuberculosis* in respiratory specimens. J Clin Microbiol.

[22] Chandran SP, Kenneth J (2010). Evaluation of COBAS TaqMan real time PCR assay for the diagnosis of *Mycobacterium tuberculosis*. Indian J Med Res.

[23] Yang YC, Lu PL, Huang SC, Jenh YS, Jou R, Chang TC (2011). Evaluation of the Cobas TaqMan MTB test for direct detection of *Mycobacterium tuberculosis* complex in respiratory specimens. J Clin Microbiol.

[24] Horita N, Narita A, Ikeda M, Nakashima K, Ushio R, Watanabe H (2016). Sensitivity and specificity of Cobas TaqMan MTB real-time polymerase chain reaction for culture-proven *Mycobacterium Tuberculosis*: meta-analysis of 27431 specimens from 19 studies. Am J Respir Crit Care Med.

[25] Notomi T, Okayama H, Masubuchi H, Yonekawa T, Watanabe K, Amino N, Hase T (2000). Loop-mediated isothermal amplification of DNA. Nucleic Acids Res.

[26] Parida M, Sannarangaiah S, Dash PK, Rao PVL, Morita K (2008). Loop mediated isothermal Amplifcation (LAMP): a new generation of innovative gene amplifcation technique; perspectives in clinical diagnosis of infectious diseases. Rev Med Virol.

[27] Iwamoto T, Sonobe T, Hayashi K (2003). Loop-mediated isothermal amplification for direct detection of *Mycobacterium tuberculosis* complex, *M. avium*, and *M. intracellulare* in sputum samples. J Clin Microbiol.

[28] Pandey BD, Poudel A, Yoda T, Tamaru A, Oda N, Fukushima Y, Lekhak B (2008). Development of an in-house loop-mediated isothermal amplification (LAMP) assay for detection of *Mycobacterium tuberculosis* and evaluation in sputum samples of Nepalese patients. J Med Microbiol.

[29] Zhu RY, Zhang KX, Zhao MQ, Liu YH, Xu YY, Ju CM, Li B, Chen JD (2009). Use of visual loop-mediated isothermal amplification of *rimM* sequence for rapid detection of *Mycobacterium tuberculosis* and *Mycobacterium bovis*. J Microbiol Methods.

[30] Aryan E, Makvandi M, Farajzadeh A, Huygen K, Bifani P, Mousavi SL, Fateh A, Jelodar A, Gouya MM, Romano M (2010). A novel and more sensitive loop-mediated isothermal amplification assay targeting *IS6110* for detection of *Mycobacterium tuberculosis* complex. Microbiol Res.

[31] Bi A, Nakajima C, Fukushima Y, Tamaru A, Sugawara I, Kimura A (2012). A rapid loop-mediated isothermal amplification assay targeting *hspX* for the detection of *Mycobacterium tuberculosis* complex. Jpn J Infect Dis.

[32] Balne PK, Barik MR, Sharma S, Basu S (2013). Development of a loop-mediated isothermal amplification assay targeting the *mpb64* gene for diagnosis of intraocular tuberculosis. J Clin Microbiol.

[33] Nimesh M, Joona D, Varma-Basilb M, Salujaa D (2014). Development and clinical evaluation of *sdaA* loop-mediated isothermal amplification assay for detection of *Mycobacterium tuberculosis* with an approach to prevent carryover contamination. J Clin Microbiol.

[34] Saharan P, Dhingolia S, Khatri P, Duhan JS, Gahlawat SK (2014). Loop-mediated isothermal amplification (LAMP) based detection of bacteria: a review. Afr J Biotechnol.

[35] Bentaleb EM, Abid M, El Messaoudi MD, Lakssir B, Ressami EM, Amzazi S, Sefrioui H, Ait Benhassou H (2016). Development and evaluation of an in-house single step loop-mediated isothermal amplification (SS-LAMP) assay for the detection of *Mycobacterium tuberculosis* complex in sputum samples from Moroccan patients. BMC Infect Dis.

[36] Ou X, Li Q, Xia H, Pang Y, Wang S, Zhao B, Song Y, Zhou Y, Zheng Y (2014). Diagnostic accuracy of the PURE-LAMP test for pulmonary tuberculosis at the county-level laboratory in China. PLoS One.

[37] Bojang AL, Mendy FS, Tientcheu LD, Out J, Antonio M, Kampmann B, Agbla S, Sutherland JS (2016). Comparison of TB-LAMP, GeneXpert MTB/RIF and culture for diagnosis of pulmonary tuberculosis in the Gambia. I infect.

[38] Gray CM, Katamba A, Narang P, Giraldo J, Zamudio C, Joloba M, Narang R, et al. Feasibility and operational performance of TB LAMP in decentralized settings -results from a multi-center study. J Clin Microbiol. 2016; 10.1128/JCM.03036-15. 10.1128/JCM.03036-15 PMC4963503 27194691

[39] Kaku T, Minamoto F, D'Meza R, Morose W, Boncy J, Bijou J, Geffrard H, Yoshida M, Mori T (2016). Accuracy of LAMP-TB method for diagnosing tuberculosis in Haiti. Jpn J Infect Dis.

[40] Ghosh PK, Chakraborty B, Maiti PK, Ray R (2017). Comparative evaluation of loop-mediated isothermal amplification and conventional methods to diagnose extrapulmonary tuberculosis. Ann Trop Med Public Health.

[41] World Health Organization (WHO). The use of Loop-mediated Isothermal Amplification (TB-LAMP) for the diagnosis of pulmonary tuberculosis: Policy guidance. 2016. ISBN 9789241511186. 27606385

[42] Blakemore R, Story E, Helb D, Kop J, Banada P, Owens MR (2010). Evaluation of the analytical performance of the Xpert MTB/RIF assay. J Clin Microbiol.

[43] Cepheid. Brochure: Xpert® MTB/RIF. Two-hour detection of MTB and resistance to rifampicin. http://www.cepheid.com/en/component/phocadownload/category/2-healthcare-impact?download=58:xpert-mtbrif-brochure-eu-0089-02-lor ˙Broch ˙R9 ˙EU.pdf. 2012. Sunnyvale, Accessed 17 June 2012.

[44] Steingart KR, Schiller I, Horne DJ, Pai M, Boehme CC, Dendukuri N (2014). Xpert MTB/RIF assay for pulmonary tuberculosis and rifampicin resistance in adults. (Review) Cochrane Database Syst Rev.

[45] World Health Organization (WHO) (2013). Automated real-time nucleic acid amplification technology for rapid and simultaneous detection of tuberculosis and rifampicin resistance: Xpert MTB/RIF system for the diagnosis of pulmonary and extrapulmonary TB in adults and children. WHO/HTM/TB/2013.14.

[46] US Food and Drug Administration. FDA permits marketing of first U.S. test labeled for simultaneous detection of tuberculosis bacteria and resistance to the antibiotic rifampin. Available at: 2014. https://wayback.archive-it.org/7993/20170112223020/http://www.fda.gov/NewsEvents/Newsroom/PressAnnouncements/ucm362602.htm. Accessed 13 Jan 2014. 24195114

[47] World Health Organization (WHO). Xpert MTB/RIF assay for the diagnosis of pulmonary and extrapulmonary TB in adults and children: Policy update. Available at: http://www.who.int/tb/publications/xpert-mtb-rif-assay-diagnosis-policy-update/en/ Accessed 15 Dec 2015.

[48] Iram S, Zeenat A, Hussain S, Yusuf NW, Askam M (2015). Rapid diagnosis of tuberculosis using Xpert MTB/RIF assay- report from a developing country. Pak J Med Sci.

[49] Lombardi G, Di Gregori V, Girometti N, Tadolini M, Bisognin F, Dal Monte P (2017). Diagnosis of smear-negative tuberculosis is greatly improved by Xpert MTB/RIF. PLoS One.

[50] Ozkutuk N, Surucüoglu S (2014). Evaluation of the Xpert MTB/RIF assay for the diagnosis of pulmonary and extrapulmonary tuberculosis in an intermediate-prevalence setting. Mikrobiyol Bul.

[51] Nicol MP, Workman L, Isaacs W (2011). Accuracy of the Xpert MTB/RIF test for the diagnosis of pulmonary tuberculosis in children admitted to hospital in cape town, South Africa: a descriptive study. Lancet Infect Dis.

[52] Rachow A, Clowes P, Saathoff E, Mtafya B, Michael E, Ntinginya EN (2012). Increased and expedited case detection by Xpert MTB/RIF assay in childhood tuberculosis: a prospective cohort study. Clin Infect Dis.

[53] Sanchez-Padilla E, Merker M, Beckert P, Jochims F, Dlamini T, Kahn P (2015). Detection of drug-resistant tuberculosis by Xpert MTB/RIF in Swaziland. N Engl J Med.

[54] André E, Goeminne L, Colmant A, Beckert P, Niemann S, Delmee M (2017). Novel rapid PCR for the detection of Ile491Phe rpoB mutation of Mycobacterium tuberculosis, a rifampicin-resistance-conferring mutation undetected by commercial assays. Clin Microbiol Infect.

[55] Mathys V, van de Vyvere M, de Droogh E, Soetaert K, Groenen G (2014). False-positive rifampicin resistance on Xpert(R) MTB/RIF caused by a silent mutation in the rpoB gene. Int J Tuberc Lung Dis.

[56] Luetkemeyer AF, Firnhaber C, Kendall MA, Wu X, Mazurek GH, Benator DA (2016). Evaluation of Xpert MTB/RIF versus AFB smear and culture to identify pulmonary tuberculosis in patients with suspected tuberculosis from low and higher prevalence settings. Clin Infect Dis.

[57] Parcell BJ, Jarchow-MacDonald AA, Seagar AL, Laurenson IF, Prescott GJ, Lockhart M (2017). Three year evaluation of Xpert MTB/RIF in a low prevalence tuberculosis setting: a Scottish perspective. J Inf Secur.

[58] Chakravorty S, Simmons AM, Rowneki M (2017). The new Xpert MTB/RIF ultra: improving detection of *Mycobacterium tuberculosis* and resistance to rifampin in an assay suitable for point-of-care testing. MBio.

[59] Alland MRD, Smith L, Ryan JJ, Chancellor M, Simmons AM, Persing D, Kwiatkowski R, Jones M, Chakravorty S (2015). Xpert MTB/RIF Ultra: A New Near-Patient TB Test With Sensitivity Equal to Culture.

[60] FIND report on accuracy study of the Ultra assay - FIND [Internet]. FIND. 2017: https://www.finddx.org/publication/ultra-report/.

[61] Dorman SE, Schumacher SG, Alland D (2018). Xpert MTB/RIF ultra for detection of *Mycobacterium tuberculosis* and rifampin resistance: a multicentre diagnostic accuracy study. Lancet Infect Dis.

[62] Arend SM, van Soolingen D (2018). Performance of Xpert MTB/RIF ultra: a matter of dead or alive. Lancet Infect Dis.

[63] Theron G, Venter R, Calligaro G (2016). Xpert MTB/RIF results in patients with previous tuberculosis: can we distinguish true from false positive results?. Clin Infect Dis.

[64] Zar H, Workman L, Nicol M (2017). Diagnosis of pulmonary tuberculosis in HIV-infected and uninfected children using Xpert MTB/RIF ultra. Am J Respir Crit Care Med.

[65] Nicol MP, Workman L, Prins M, Bateman L, Ghebrekristos Y, Mbhele S, et al. Accuracy of Xpert MTB/RIF ultra for the diagnosis of pulmonary tuberculosis in children. Pediatr Infect Dis J. 2018; 10.1097/INF.0000000000001960. 10.1097/INF.0000000000001960 29474257

[66] World Health Organization (WHO). Next-generation Xpert® MTB/RIF Ultra assay recommended by WHO. 2017. http://who.int/tb/features_archive/Xpert-Ultra/en/.

[67] García-Basteiro AL, Di Nardod A, .Saavedraa B, Silva DR, et al. Point of care diagnostics for tuberculosis. Rev Port Pneumol 2018; 10.1016/j.rppnen.2017.12.002.

[68] Denkinger C (2015). The TB diagnose pipeline.

[69] Monedero I, Bhavaraju R, Mendoza-Ticona A, Sánchez-Montalvá A. The paradigm shift to end tuberculosis. Are we ready to assume the changes? Expert Rev Respir Med. 2017; 10.1080/17476348.2017.1335599. 10.1080/17476348.2017.1335599 28562103

[70] MacLean E, Huddart S, Pai M. Molecular diagnosis of tuberculosis: we need solutions that span the healthcare value chain. Expert Rev Mol Diagn. 2017;17(1) 10.1080/14737159.2017.1265889 27892734

[71] De Beenhouwer H, Lhiang Z, Jannes G, Mijs W, Machtelinckx L, Rossau R (1995). Rapid detection of rifampin resistance in sputum and biopsy specimens from tuberculosis patients by PCR and line probe assay. Tuber Lung Dis.

[72] WHO. Policy statement. Molecular line probe assays for rapid screening of patients at risk of multidrug resistant tuberculosis (MDR-TB). 2008. (http://www.stoptb.org/assets/documents/about/cb/meetings/15/2.08-11%20Rolling%20out%20diagnostics%20in%20the%20field/2.08-11.2%20Line%20Probe%20Assays.pdf. Accessed 18 Nov 2009).

[73] WHO. The use of molecular line probe assays for the detection of resistance to isoniazid and rifampicin: policy update. Geneva: 2016 update. Geneva: (WHO/HTM/TB/2016.12; http://apps.who.int/iris/bitstream/10665/250586/1/9789241511261-eng.pdf?ua=1, accessed 1 June 2017).

[74] Somoskovi A, Dormandy J, Rivenburg J, Pedrosa M, McBride M, Max Salfinger M (2008). Direct comparison of the GenoType MTBC and genomic deletion assays in terms of ability to distinguish between members of the *Mycobacterium tuberculosis* complex in clinical isolates and in clinical specimens. J Clin Microbiol.

[75] Albert H, Bwanga F, Mukkada S, Nyesiga B, Ademun JP, Lukyamuzi G (2010). Rapid screening of MDR-TB using molecular line probe assay is feasible in Uganda. BMC Infect Dis.

[76] Tomasicchio M, Theron G, Pietersen E (2016). The diagnostic accuracy of the MTBDR*plus* and MTBDR*sl* assays for drug-resistant TB detection when performed on sputum and culture isolates. Sci Rep.

[77] Meaza A, Kebede A, Yaregal Z, Dagne Z, Moga S, Yenew B, Desta K (2017). Evaluation of genotype MTBDR*plus* VER 2.0 line probe assay for the detection of MDR-TB in smear positive and negative sputum samples. BMC Infect Dis.

[78] Huang W, Chen H, Kuo Y, Jou R (2009). Performance assessment of the GenoType MTBDR*plus* test and DNA sequencing in detection of multidrug-resistant *Mycobacterium tuberculosis*. J Clin Microbiol.

[79] Nikolayevsky V, Balabanova Y, Simak T, Malomanova N, Fedorin I, Drobniewski F (2009). Performance of the genotype® MTBDR*plus* assay in the diagnosis of tuberculosis and drug resistance in Samara, Russian Federation. BMC Clin Pathol.

[80] Anek-Vorapong R (2010). Validation of the GenoType MTBDR*plus* assay for detection of MDR-TB in a public health laboratory in Thailand. BMC Infect Dis.

[81] Barnard M, Gey van Pittius NC, van Helden PD, Bosman M, Coetzee G, Warren RM (2012). The diagnostic performance of the GenoType MTBDR*plus* version 2 line probe assay is equivalent to that of the Xpert MTB/RIF assay. J Clin Microbiol.

[82] HAIN LifeScience. GenoType MTBDR*sl*VER 2.0 instructions for use. Document IFU-317A-01. HAIN LifeScience, Nehren, Germany. 2015; http://www.hain-lifescience.de/en/instructions-for-use.html.

[83] WHO (2016). The use of molecular line probe assays for the detection of resistance to second-line anti-tuberculosis drugs–policy guidance.

[84] Gardee Y, Dreyer AW, Koornhof HJ, Omar SV, da Silv P, Bhyat Z, Ismaila NA (2017). Evaluation of the GenoType MTBDR*sl* version 2.0 assay for second-line drug resistance detection of *Mycobacterium tuberculosis* isolates in South Africa. J Clin Microbiol.

[85] Cheng S, Cui Z, Li Y, Hu Z (2014). Diagnostic accuracy of a molecular drug susceptibility testing method for the antituberculosis drug ethambutol: a systematic review and meta-analysis. J Clin Microbiol.

[86] Simons SO, van der Laan T, de Zwaan R, Kamst M, van Ingen J, Dekhuijzen PN (2015). Molecular drug susceptibility testing in the Netherlands: performance of the MTBDR*plus* and MTBDR*sl* assays. Int J Tuberc Lung Dis..

[87] Frick M, Lessem E, Mckenna L. Drugs, Diagnostics, Vaccines, Preventive Technologies, Research Toward a cure, and immune-based and gene therapies in Development 2016 PIPELINE REPORT. Tuberculosis (TB) Edition: Diagnostics, Treatment, Prevention, and Vaccines in Development.

[88] Mitarai S, Okumura M, Toyota E, Yoshiyama T, Aono A (2011). Evaluation of a simple loop-mediated isothermal amplification test kit for the diagnosis of tuberculosis. Int J Tuberc Lung Dis..

[89] Agarwal A, Gaurav Sharma G, Dut Jasuj N (2016). Analysis of diagnostic methods and their sensitivity test for *Mycobacterium tuberculosis*. SGVU Int J Env Sci Technol.

[90] Kouzaki Y, Mikita K, Maeda T, Ishihara M, Kawano S, Fujikura Y, Imai K, Kanoh S, Utsunomiya K, Inoue M, Miyahira Y, Kawana A (2015). PURE-LAMP procedure for the diagnosis of Extrapulmonary tuberculosis: a case series. Intern Med.

[91] French DJ, Archard CL, Brown T, McDowell DG (2001). HyBeacon probes: a new tool for DNA sequence detection and allele discrimination. Mol Cell Probes.

[92] Hofmann-Thiel S, Hoffmann H (2014). Evaluation of Fluorotype MTB for detection of *Mycobacterium tuberculosis* complex DNA in clinical specimens from a low-incidence country. BMC Infect Dis.

[93] Eigner U, Veldenzer A, Holfelder M (2013). Reliable detection of *Mycobacterium Tuberculosis* in respiratory tract specimens. Clin Lab.

[94] Obasanya J, Lawson L, Edwards T, Olanrewaju O, Madukaji L, Dacombe R, Dominguez J, Molina-Moya B, Adams ER, Cuevas LE (2017). FluoroType MTB system for the detection of pulmonary tuberculosis. ERJ Open Res.

[95] Lessem E. The tuberculosis diagnostics pipeline. Pipeline report: HIV, TB, and HCV. Drugs, diagnostics, vaccines, preventive technologies, research toward a cure, and immune-based and gene therapies in Development 2017. p. 91–106.

[96] Fang R, Li X, Hu L, You Q, Li J, Wu J, Xu P, Zhong H, Luo Y, Mei J, Gao Q (2009). Cross-priming amplification for rapid detection of *Mycobacterium tuberculosis* in sputum specimens. J Clin Microbiol.

[97] UNITAID. Tuberculosis Diagnostic technology and market Landscape. 3rd ed. Geneva; 2014.

[98] Ou X, Song Y, Zhao B, Li Q, Xia H, Zhou Y, Pang Y, Wang S, Zhang Z, Cheng S, Liu C, Zhao Y (2014). A multicenter study of cross-priming amplification for tuberculosis diagnosis at peripheral level in China. Tuberculosis (Edinb).

[99] Mhimbira FA, Bholla M, Sasamalo M, Mukurasi W, Hella JJ, Jugheli L, Reither K (2015). Detection of *Mycobacterium tuberculosis* by EasyNAT diagnostic kit in sputum samples from Tanzania. J Clin Microbiol.

[100] Bholla M, Kapalata N, Masika E, Chande H, Jugheli L, Sasamalo M (2016). Evaluation of Xpert® MTB/RIF and Ustar EasyNAT™ TB IAD for diagnosis of tuberculous lymphadenitis of children in Tanzania: a prospective descriptive study. BMC Infect Dis.

[101] Barnard M, Albert H, Coetzee G, O'Brien R, Bosman ME (2008). Rapid molecular screening for multidrug-resistant tuberculosis in a high-volume public health laboratory in South Africa. Am J Respir Crit Care Med.

[102] Boehme CC, Nabeta P, Hillemann D, Nicol MP, Shenai S, Krapp F (2010). Rapid molecular detection of tuberculosis and rifampin resistance. N Engl J Med.

[103] Weyer K, Mirzayev F, Migliori GB, Van Gemert W, D'Ambrosio L (2013). Rapid molecular TB diagnosis: evidence, policy making and global implementation of Xpert MTB/RIF. Eur Respir J.

[104] Drobniewski F, Nikolayevskyy V, Maxeiner H, Balabanova Y (2013). Rapid diagnostics of tuberculosis and drug resistance in the industrialized world: clinical and public health benefits and barriers to implementation. BMC Med.

[105] Siu GK, Zhang Y, Lau TC, Lau RW, Ho PL, Yew WW, Tsui SK (2011). Mutations outside the rifampicin resistance-determining region associated with rifampicin resistance in *Mycobacterium tuberculosis*. J Antimicrob Chemother.

[106] Alonso M, Palacios JJ, Herranz M, Penedo A, Menéndez A, Bouza E (2011). Isolation of *Mycobacterium tuberculosis* strains with a silent mutation in *rpoB* leading to potential misassignment of resistance category. J Clin Microbiol.

[107] Aubry A, Sougakoff W, Bodzongo P, Delcroix G, Armand S, Millot G (2014). First evaluation of drug-resistant *Mycobacterium tuberculosis* clinical isolates from Congo revealed misdetection of fluoroquinolone resistance by line probe assay due to a double substitution T80A-A90G in *GyrA*. PLoS One.

[108] Nimmo C, Doyle R, Burgess C, Williams R, Gorton R, McHugh TD (2017). Rapid identification of a *Mycobacterium tuberculosis* full genetic drug resistance profile through whole genome sequencing directly from sputum. Int J Infect Dis.

[109] Shea J, Halse TA, Lapierre P, Shudt M, Kohlerschmidt D, Van Roey P, Limberger R, Taylor J, Escuyer V, Musser KA (2017). Comprehensive whole-genome sequencing and reporting of drug resistance profiles on clinical cases of *Mycobacterium tuberculosis* in New York state. J Clin Microbiol.

[110] Nikolayevskyy V, Kranzer K, Niemann S, Drobniewski F (2016). Whole genome sequencing of *Mycobacterium tuberculosis* for detection of recent transmission and tracing outbreaks: a systematic review. Tuberculosis.

[111] Satta G, Lipman M, Smith GP, Arnold C, Kon OM, McHugh TD. *Mycobacterium tuberculosis* and whole-genome sequencing: how close are we to unleashing its full potential? Clin Microbiol Infect. 2017; doi.org/10.1016/j.cmi.2017.10.030 10.1016/j.cmi.2017.10.030 29108952

[112] Brown AC, Bryant JM, Einer-Jensen K, Holdstock J, Houniet DT, Chan JZ (2015). Rapid whole-genome sequencing of *Mycobacterium tuberculosis* isolates directly from clinical samples. J Clin Microbiol.

[113] Coll F, McNerney R, Preston MD, Guerra-Assunção JA, Warry A (2015). Rapid determination of anti-tuberculosis drug resistance from whole-genome sequences. Genome Med.

[114] Török ME, Reuter S, Bryant J, Köser CU, Stinchcombe SV, Nazareth B (2013). Rapid whole-genome sequencing for investigation of a suspected tuberculosis outbreak. J Clin Microbiol.

[115] Ryu YJ (2015). Diagnosis of pulmonary tuberculosis: recent advances and diagnostic algorithms. Tuberc Respir Dis (Seoul).

[116] Verza M, Schmid KB, Barcellos RB, Linck N, Bello GL (2017). Performance of a molecular assay to detect *Mycobacterium tuberculosis* complex DNA in clinical specimens: multicenter study in Brazil. Mem Inst Oswaldo Cruz.

